# Mediodorsal thalamus and cognition in non-human primates

**DOI:** 10.3389/fnsys.2013.00038

**Published:** 2013-08-06

**Authors:** Mark G. Baxter

**Affiliations:** Glickenhaus Laboratory of Neuropsychology, Department of Neuroscience and Friedman Brain Institute, Icahn School of Medicine at Mount SinaiNew York, NY, USA

**Keywords:** thalamus, mediodorsal nucleus, retrograde amnesia, anterograde amnesia, hippocampus, prefrontal

## Abstract

Several recent studies in non-human primates have provided new insights into the role of the medial thalamus in different aspects of cognitive function. The mediodorsal nucleus of the thalamus (MD), by virtue of its connectivity with the frontal cortex, has been implicated in an array of cognitive functions. Rather than serving as an engine or relay for the prefrontal cortex, this area seems to be more specifically involved in regulating plasticity and flexibility of prefrontal-dependent cognitive functions. Focal damage to MD may also exacerbate the effects of damage to other subcortical relays. Thus, a wide range of distributed circuits and cognitive functions may be disrupted from focal damage within the medial thalamus (for example as a consequence of stroke or brain injury). Conversely, this region may make an interesting target for neuromodulation of cognitive function via deep brain stimulation or related methods, in conditions associated with dysfunction of these neural circuits.

## Introduction

Midline thalamic nuclei are key elements of distributed neural circuits for many different aspects of cognitive function. The goal of this mini-review is to discuss several recent studies of the roles of one of these nuclei, the mediodorsal nucleus (MD), in cognitive function. In these studies, neurotoxic lesion experiments in non-human primates have suggested that MD is not simply a relay nucleus to/from the frontal cortex, but plays a distinct role in modulation of cognitive functions to the extent that damage to MD does not simply mimic the effects of damage to frontal cortex.

One goal of experimental lesion studies within the medial thalamus has been to dissect the neuropathological basis of diencephalic amnesia, following strokes or traumatic injuries to the thalamus and adjacent structures, or in conditions such as alcoholic Korsakoff syndrome. These have identified the contributions of multiple regions to various aspects of cognitive function, including anterior thalamic nuclei, the mammillary bodies, as well as MD. Impairments in executive function have also been reported following damage to the thalamus in humans, implicating thalamic nuclei in neural circuits beyond those critical for memory. Because monkeys exhibit a complex array of cognitive behaviors, and have a well-differentiated prefrontal cortex, they have been particularly useful models to help understand the functions of MD, with regards to its role in both memory and behavioral control by the prefrontal cortex.

## Mediodorsal thalamus and retrograde/anterograde amnesia

It is doubly difficult to understand the necessity of specific brain regions for memory consolidation and retrieval based on studies in human patients, because the lesions are rarely specific to a particular brain structure, and because levels of pre-injury memory have not been measured. Lesion-based symptom mapping and clever neuropsychological assessments can offset these problems to some degree, but lesion studies in animals provide an important complementary approach because both brain damage and pre-injury memory are under experimental control. Analyses in humans with thalamic damage have implicated the mammillothalamic tract, in particular, in impaired formation of new memories (anterograde amnesia) (Van der Werf et al., 2000, 2003). Individuals with Korsakoff syndrome (and widespread diencephalic degeneration) have extensive impairment in retrieval of memories formed before brain damage was sustained (retrograde amnesia), but retrograde amnesia is not associated with focal lesions of the thalamus (Kopelman et al., 1999, 2009). An initial investigation in monkeys reported no retrograde amnesia after focal neurotoxic lesions of the medial part of MD, but a significant anterograde amnesia (Mitchell and Gaffan, 2008), congruent to some degree with the clinical literature.

The focus on medial MD reflects its connectivity with the frontal cortex and networks involved in memory and cognitive function more generally. Four subdivisions of MD are recognized in the primate brain: the medial, magnocellular part (MDmc), a more lateral parvicellular part (MDpc), the most lateral multiformis portion (MDmf) that forms a band at the lateral edge of MD, and the densocellular portion (MDde) located caudally lateral to the MDpc and habenula (Goldman-Rakic and Porrino, 1985). The medial, magnocellular division may be further subparcellated (Ray and Price, 1993). Ventrolateral and orbital prefrontal cortex predominantly receive input from neurons in medial MD (MDmc) whereas dorsolateral prefrontal cortex mainly receives input from more lateral MD (MDpc) (Goldman-Rakic and Porrino, 1985; Barbas et al., 1991). MD projections to the prefrontal cortex mainly target cortical layers III and IV, whereas projections back to the MD from prefrontal cortex originate from deep layers V and VI (Giguere and Goldman-Rakic, 1988; Xiao et al., 2009). The medial MD also receives input from the amygdala (Porrino et al., 1981) and rhinal cortex (Russchen et al., 1987; Goulet et al., 1998) and projects to the thalamic reticular nucleus, a key node for gating thalamocortical interaction (Zikopoulos and Barbas, 2012). The role of the MD in memory has been viewed mainly through interactions with amygdala/rhinal cortex and frontal cortex (Gaffan and Murray, 1990; Gaffan et al., 1993). The neurotoxic lesions of medial MD whose behavioral effects were investigated in the studies described below produced extensive damage to medial, magnocellular MD (MDmc) as well as unavoidable damage to midline thalamic nuclei located between the two halves of MD, including the rhomboid, centromedian, and paraventricular nucleus. Damage to these midline nuclei on their own cannot account for the behavioral effects of neurotoxic medial MD lesions (Gaffan and Murray, 1990; Mitchell and Gaffan, 2008) but could exacerbate the effects of bilateral MDmc damage.

We carried out a follow-up study to further explore the role of subcortical structures, including the thalamus, vs. cortical structures in retrograde and anterograde amnesia, using the same kind of stimulus material as Mitchell and Gaffan (2008). The stimulus material was object-in-place scene problems (Gaffan, 1994). These stimuli are presented on a large touch-sensitive screen and composed of a randomly colored background, a random number of randomly colored ellipse segments, a single large typographical (ASCII) character, and two small typographical characters. The two small characters are the “objects” and the remaining visual elements constitute the “scene.” Monkeys are taught that within each scene, one of the two objects is correct (a touch to that object generates a reward) whereas the other is incorrect (a touch to that object generates no reward); a touch to any other element of the scene causes the screen to blank and the trial to repeat after a brief interval. Rhesus monkeys learned three sets of 100 object-in-place scene problems preoperatively, in sequential order, to a 90% performance criterion. They then received a single-trial retention test on each scene and were assigned to surgical groups balanced for preoperative performance. Postoperatively, each monkey received another single-trial retention test, received a number of retraining sessions on the preoperatively-learned scenes, and learned a new set of 100 scenes. The single-trial retention tests allowed for a sensitive within-subject measure of the degree of retrograde amnesia, and the postoperative acquisition of a new set of scenes allowed for a measure of anterograde amnesia with the same stimulus material. In the followup study, we tested two groups of monkeys (as well as an unoperated control group): one with focal ablations of the anterior entorhinal cortex, and one with neurotoxic lesions of the medial MD combined with transection of the fornix. This second group was intended to produce a widespread disconnection of subcortical networks involved in memory, including both projections from medial, magnocellular MD to ventrolateral and orbital prefrontal cortex (Goldman-Rakic and Porrino, 1985) and subcortical connections of the hippocampus (including, but not limited to, with the mammillary bodies). We found that entorhinal cortex lesions produced retrograde but not anterograde amnesia, whereas MD + fornix lesions produced both retrograde and anterograde amnesia (Mitchell et al., 2008).

**Figure 1:**
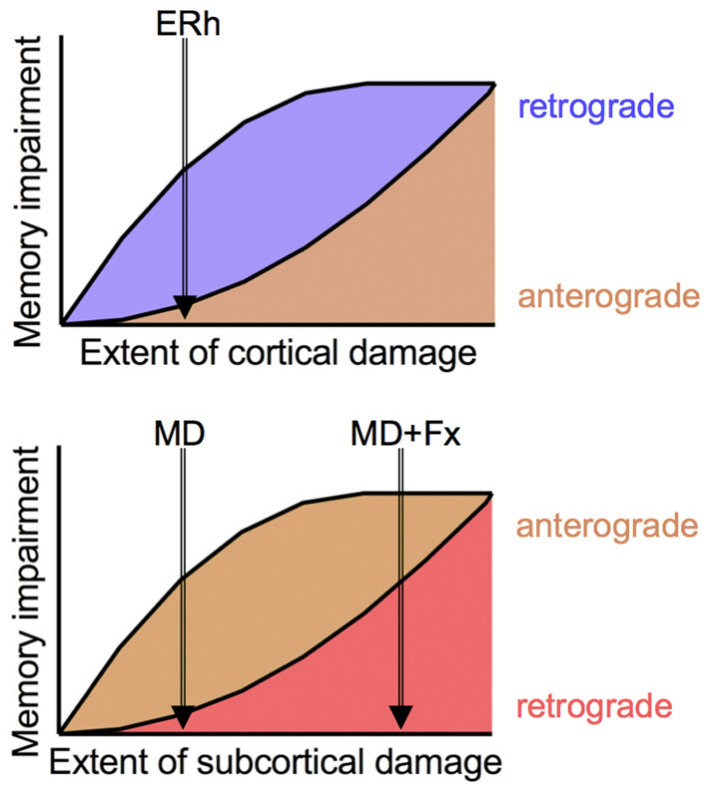
**Conceptual model of relationship between cortical and subcortical damage and degree of retrograde and anterograde amnesia.** As degree of damage increases, so does severity of amnesia, but retrograde amnesia is more sensitive to cortical damage (**Top panel**) and anterograde amnesia is more sensitive to subcortical damage (**Bottom panel**). Small cortical lesions, as of anterior entorhinal cortex (ERh), produce retrograde amnesia but no anterograde amnesia; small subcortical lesions, as neurotoxic lesions of medial MD (MD), produce anterograde amnesia but no retrograde amnesia. More extensive subcortical lesions, as of MD and fornix (MD + Fx) produce both anterograde and retrograde amnesia.

Taken together with the earlier result with neurotoxic medial MD lesions using a slightly different test procedure (Mitchell and Gaffan, 2008) and other findings on cortical lesions and retrograde amnesia (e.g., Thornton et al., 1997) these findings suggest a general model in which subcortical damage primarily contributes to anterograde amnesia whereas cortical damage primarily contributes to retrograde amnesia (Mitchell et al., 2008). Of course, in the limit, extensive cortical or subcortical damage would be expected to produce both kinds of amnesia. Presumably this accounts for the combination of retrograde and anterograde amnesia observed after medial MD + fornix lesions, as well as for the complex patterns of retrograde and anterograde amnesia in humans after brain lesions that likely affect both cortical and subcortical areas, either by direct damage or by virtue of interruption of fibers of passage traveling adjacent to or through lesioned cortex. On this view, formation of new memories is much more sensitive to subcortical damage (anterograde amnesia) and retrieval of old memories is much more sensitive to cortical damage (retrograde amnesia), although the degree of both kinds of amnesia increases as the amount of brain damage increases (Figure 1). This may help explain, for example, why focal thalamic lesions tend not to cause retrograde amnesia, but the more widespread damage that occurs in Korsakoff's syndrome is associated with both retrograde and anterograde amnesia.

## Modulation of prefrontal function by mediodorsal thalamus

A previous study of medial MD lesions in monkeys, produced by direct surgical aspiration, concluded that the involvement of MD in memory reflected a disruption of prefrontal cortex function (Gaffan and Parker, 2000) because monkeys with aspiration lesions of medial MD were impaired in both scene learning and in acquisition of object-reward association problems, and this more generalized deficit is not seen in monkeys with lesions of the fornix-mammillary system or disconnection of prefrontal and inferotemporal cortex (Gaffan et al., 2001, 2002). Thus, medial MD ablations may have caused a widespread disruption of prefrontal cortex function, resembling effects of bilateral prefrontal damage (Parker and Gaffan, 1998). Based on the failure of neurotoxic lesions of medial MD to cause extensive retrograde amnesia (Mitchell and Gaffan, 2008), this issue was ripe for re-examination with neurotoxic lesions of medial MD. Although data from bilateral prefrontal lesions in the same retrograde amnesia paradigm are not available, it is worth noting that prefrontal-inferotemporal disconnection produces extensive retrograde amnesia for some kinds of visual discrimination problems that can be learned normally postoperatively (Browning and Gaffan, 2008), implicating the prefrontal cortex, as well, in some aspects of memory retrieval.

One approach to this question was to compare performance of monkeys with neurotoxic medial MD lesions on learning new scene memory problems, in this case a set of 20 problems presented within a testing day rather than sets of 100 problems learned across days (to facilitate comparison with earlier studies of aspiration lesions of medial MD), with performance on a complex strategy implementation task that is impaired by disconnection of frontal and inferotemporal cortex but not by extensive lesions of temporal lobe white matter tracts (Gaffan et al., 2002). In this task, monkeys learn about two classes of stimuli (cliparts), each class associated with a different strategy for obtaining reward. The first class, “persistent” (P), requires four consecutive choices of a P object on four consecutive trials in order to earn a reward. At any time after earning a reward for four consecutive P choices, a touch to the second class of stimuli, “sporadic” (S), generates a reward immediately, but another reward cannot be earned for choosing an S object until another reward is earned for four consecutive P choices. There are four pairs of these stimuli, each containing one P and one S stimulus, and they are randomly intermixed across trials. Thus, the monkey's optimal strategy is to choose PPPPSPPPPSPPPPS … across trials, neither interrupting sequences of P choices with an S choice before reward is earned nor continuing to choose S when a reward has already been earned for choosing S (and another reward for four consecutive P choices has not been earned yet). The ratio of trials worked to number of rewards earned provides a summary measure of how effective their implementation of the strategies is. Monkeys learn to perform this task very well, close to the perfect trials/reward ratio of 2.5, but their performance becomes disorganized after surgical disconnection of frontal and temporal cortex, such that the trials/reward ratio greatly increases, indicating the monkeys are not applying the choice strategies appropriately. Moreover, their behavior consists of both inappropriate S responses and a failure to make appropriate S responses as soon as they would be rewarded, so their deficit is not simply a failure to inhibit responding to the more attractive S stimulus (Gaffan et al., 2002; Baxter et al., 2009). Within the prefrontal cortex, performance on this task is significantly impaired by bilateral ablations of ventrolateral prefrontal cortex but not dorsolateral or orbital prefrontal cortex (Baxter et al., 2007, 2008b, 2009). Thus, loss of projections from medial MD to ventrolateral prefrontal cortex might be expected to impair performance on the strategy implementation task, if these projections are generally necessary for normal prefrontal cortex function.

Rhesus monkeys were preoperatively trained on both these tasks (object-in-place scene learning and strategy implementation), given a preoperative performance test, and received neurotoxic lesions of medial MD, followed by a postoperative performance test after post-surgical recovery. Remarkably, neurotoxic lesions of medial MD impaired scene learning to a similar degree as aspiration lesions, but were without significant effect on the strategy implementation task (Mitchell et al., 2007a). This indicates that damage to medial MD does not generally impair the function of its prefrontal targets. It also confirms the anterograde amnesia observed after medial MD lesions (Mitchell and Gaffan, 2008) extends to rapid, within-session learning of scenes, which also depends on an intact prefrontal cortex (Browning et al., 2005; Baxter et al., 2007, 2008a). We have proposed that the advantage in speed of learning conveyed by the unique background scenes (Gaffan, 1994) reflects the involvement of the prefrontal cortex in generating retrieval cues based on the unique background scenes to bridge successive presentations of the problems, creating an element of temporal complexity to this task that is not present in discrimination learning without unique background stimuli (Wilson et al., 2010). Thus, the MD may be involved in regulating plasticity within prefrontal cortex as these cues are acquired and generated. This loss of plasticity causes anterograde amnesia, while sparing execution of well-learned retrieval cues and behavioral strategies, allowing unimpaired retention of preoperatively learned scenes and performance on the strategy implementation task. Notably, frank prefrontal damage impairs all these behavioral domains, underlining the distinction between effects of selective MD damage and damage to the prefrontal cortex. The more discrete effects of neurotoxic lesions of medial MD, relative to aspirations of medial MD (Gaffan and Parker, 2000), presumably reflect damage to fibers of passage through medial MD caused by aspiration of the structure, perhaps including other divisions of MD and other regions of the thalamus.

Further insight into the functions of MD comes from a study of goal-directed choice behavior (Mitchell et al., 2007b). In this study, monkeys with neurotoxic lesions of medial MD learned a large set of object-reward association problems, in which half the rewarded objects (cliparts presented on a touchscreen) were rewarded with one distinct food (a half-peanut) whereas the others were rewarded with a different food (an M&M). Monkeys with neurotoxic lesions of medial MD acquired these problems at an equivalent rate to controls, again underscoring the selectivity of their memory impairment relative to that caused by aspiration lesions of medial MD (Gaffan and Parker, 2000). They were then confronted with sessions of critical trials in which they chose between pairs of rewarded objects, one peanut-rewarded and one M&M-rewarded, composed of randomly re-pairing the rewarded objects from the discrimination problems. Before some of these sessions, monkeys were satiated on one of the two food rewards: they were allowed to consume as much of that food as they could before being brought to the touchscreen testing apparatus. Performance on these “devaluation” sessions was compared to baseline performance in the critical trials. Normal monkeys will adjust their choice behavior in devaluation sessions, avoiding choices of objects associated with the devalued food. Because they encounter each object only once in each critical trial session they do not have the opportunity to learn new associations between objects and the current value of the reward, so they must rely on their representation of the expected outcome of their choice in order to guide behavior. Devaluation performance is disrupted by lesions of orbital prefrontal cortex, amygdala, or surgical disconnection of these two structures (Málková et al., 1997; Baxter et al., 2000, 2009; Izquierdo et al., 2004) but not by damage to dorsolateral or ventrolateral prefrontal cortex (Baxter et al., 2008b, 2009). Monkeys with neurotoxic MD lesions were mildly, but significantly, impaired in their devaluation performance (Mitchell et al., 2007b). Surgical disconnections of medial MD from amygdala and orbital prefrontal cortex confirm that the participation of this structure in devaluation is via interaction with these two structures (Izquierdo and Murray, 2010).

This result suggests that projections from the medial MD to orbital prefrontal cortex may play a role in updating representations of expected outcomes of choices when the values of those outcomes change, in this case because of a change in the value of the food reward as a consequence of devaluation. Like object-in-place scene learning, this reflects a form of plasticity within prefrontal cortex, as compared to the retention of preoperatively learned scenes, the implementation of a well-learned strategy as in the strategy implementation task, or the gradual acquisition of associative strength by visual stimuli that presumably can be represented outside prefrontal cortex, as in the case of object-reward association learning.

## Implications for future work and therapeutics

Taken together, these experiments indicate that MD cannot simply be regarded as a relay nucleus for information to reach the prefrontal cortex, or a general source of modulation that supports all behavioral functions of the prefrontal cortex. Both of these points of view would imply a much greater correspondence between the effects of MD damage and prefrontal cortex damage than is observed experimentally. These data instead imply a role for MD in representational plasticity within prefrontal cortex, which would encompass some aspects of memory as well as dysexecutive syndromes associated with MD damage in humans (Van der Werf et al., 2000, 2003), a point of view supported by some related research with rats (Chudasama et al., 2001; Pickens, 2008).

This raises the possibility that neuromodulation of medial MD, for example via deep brain stimulation approaches, might be a potential target for improvement of prefrontal function in neuropsychiatric conditions and other disorders of cognition. A recent report of synchronization in the beta range between MD and frontal cortex in mice (Parnaudeau et al., 2013) during performance of a working memory task is congruent with our evidence for a critical role of MD-prefrontal interaction in cognition in non-human primates, and supports the notion that neuromodulation of MD may be therapeutic when prefrontal cortex function is impaired.

### Conflict of interest statement

The author declares that the research was conducted in the absence of any commercial or financial relationships that could be construed as a potential conflict of interest.
